# Estimation of Inhaled Effective Doses of Uranium and Thorium for Workers in Bayan Obo Ore and the Surrounding Public, Inner Mongolia, China

**DOI:** 10.3390/ijerph18030987

**Published:** 2021-01-22

**Authors:** Yao Zhang, Xianzhang Shao, Liangliang Yin, Yanqin Ji

**Affiliations:** China CDC Key Laboratory of Radiological Protection and Nuclear Emergency, Chinese Center for Disease Control and Prevention, National Institute for Radiological Protection, Beijing 100088, China; paranow@163.com (Y.Z.); Shaoxianz@126.com (X.S.); yinliangliang@nirp.chinacdc.cn (L.Y.)

**Keywords:** occupational exposure, particulate matter, radioactive elements, inhaled effective dose

## Abstract

Uranium and thorium are two common natural radioactive elements with high concentrations in Earth’s crust. The main aim of this study is to estimate the inhaled effective dose of uranium and thorium caused by a typical radioactive rare earth ore to the occupational population and the surrounding public. The particulate matter (PM) concentrations in the atmosphere of four typical workplaces and one surrounding living area were obtained by a high-flow sampling equipment with a natural cellulose filter membrane. The critical parameter for the inhaled effective dose estimation—the activity median aerodynamic diameter (AMAD)—was determined. The AMAD values of uranium and thorium in the atmosphere PM were 3.36 and 3.64 μm, respectively. The estimated median effective dose caused by inhalation thorium among the occupational population ranged from 15.3 to 269.0 μSv/a, and the corresponding value for the surrounding public was 2.3 μSv/a. All values for the effective dose caused by the inhalation of uranium were in the nSv magnitude.

## 1. Introduction

Radiological hazards in industries involving naturally occurring radioactive material are a widespread radioactive problem and thus have attracted the attention of many researchers [1,2]. Uranium and thorium are two of the most enriched natural radioactive elements in Earth’s crust and thus represent a high concern. Uranium and thorium are both radioactive and chemical toxicants and can cause leukemia, renal and nervous system diseases, cancer, and so on [3,4]. Ingestion and inhalation are the two main pathways for uranium and thorium entering the body. However, inhalation is the dominant intake route for places with high particulate matter (PM) concentration in the atmosphere. Therefore, the knowledge of uranium and thorium concentrations in the atmosphere is important for evaluation of inhaled effective doses, which can predict their radiological hazard to the population [5]. The inhaled uranium and thorium continue to decay until a stable nuclide is formed. In this process, ionizing radiation that induces biological damage in human organs is emitted [5]. The main biological effects for such ionizing radiation were heritable diseases and cancer [6,7]. It has also been reported that the total risk of the above-mentioned effects for adults was 4.2 × 10^−2^/Sv [7]. Past epidemiological studies [8] showed that the elevated standard mortality ratio (SRM) due to pancreatic ailments among 592 workers exposed to thorium PM was 4.13. Moreover, a 30-year follow-up study consisting of 995 workers from a uranium-processing facility revealed a significant increase in death due to various causes [9].

The ore mine in Bayan Obo, as selected in this study, is a typically associated radioactive rare earth (RE) mine with high radiation levels. The maximum values of the external radiation dose for this ore area and the surrounding living area were previously reported as 1184 and 172 nGy/h, respectively [10]; these values are higher than the world average level (57 nGy/h) [11]. The average thorium concentration in this RE ore is approximately 0.04% [12], which is nearly 50 times higher than that in the earth’s crust [11]. The present studies concerning the radioactivity of radioactive mines have mainly focused on the ionizing radiation dose rate of different workplaces, the radionuclide contents of the ore, radon concentrations in the atmosphere, and so on [10,13,14]. However, the data are limited in terms of the estimation of internal doses during ore occupational activities as a result of the inhalation of radioactive uranium and thorium in the atmosphere.

PM size and its concentration in the atmosphere are the two important factors for determining their potential hazard to the human body [15]. The activity median aerodynamic diameter (AMAD) is the most commonly used parameter to describe PM size. The previous studies [16,17] showed that AMAD is a dominating factor for determining pulmonary deposition. The smaller the PM, the easier it is to deposit in smaller airways; the larger is the PM, the easier it is to deposit in the larger airways. Besides, AMAD is a critically assumed parameter for estimating the effective dose of inhaled radionuclide. Therefore, determining the AMAD of PM and the atmospheric concentration when the inhaled effective dose is estimated is necessary.

The RE production activity of ore manifests potential radioactive hazard to occupational workers and the surrounding public. During ore mining activities, inhalation is the main pathway for uranium and thorium PM to enter the human body. Therefore, the inhaled effective dose of workers and the surrounding public require great attention. In China, the atmospheric concentrations of uranium and thorium of an associated radioactive area and their PM sizes are rarely investigated. Thus, this study aims to estimate the effective dose of uranium and thorium inhaled by humans in the workplace and the surrounding area.

## 2. Materials and Methods

### 2.1. Sample Collection

All samples were collected using natural cellulose filter membranes. In view of estimating the inhaled effective dose of workers and the surrounding public, the samples were obtained from four typical workplaces and one surrounding living area by using high-flow sampling equipment. The four typical working areas are located in the ore site of Bayan Obo, while the surrounding living area is approximately 4 km away from this ore site. A total of 39 samples (31 samples from the workplaces and 8 samples from the surrounding living area) were obtained. The sampling flow rate was 2.0 m^3^/min, and the height of the sampling detector was approximately 1.2 m from the ground. The sampling time in the workplaces was half an hour due to the high PM concentration in these areas, while time in the surrounding living area was 6 h. Both sampling time and sampling location were recorded. The collected samples were carefully placed in numbered plastic bags after sampling.

As a means of determining the AMAD of uranium and thorium PM in the living area, the samples were acquired using a size-segregated sampling equipment. The sampling filter membranes were classified into the following six grades: 0.39–0.69, 0.69–1.3, 1.3–2.1, 2.1–4.2, 4.2–10.2, and >10.2 µm. The collecting time was 12 h per sample, and the sampling flow rate was maintained at 0.57 m^3^/min. A total of 37 samples involving 222 filter membranes were obtained. However, workplace AMAD is still assumed to be the value recommended in a previous report [18].

### 2.2. Apparatus and Reagents

The inductively coupled plasma mass spectrometry (ICP-MS) apparatus used in this research was Element 2 (Thermo Finnigan, Bremen, Germany), which is commonly applied to the sector field with high resolution. A high-volume and size-segregated sampler (IFIA 2, Staplex, New York, NY, USA; Model 236, Staplex, New York, NY, USA) and its corresponding cellulose filter membranes (Staplex, New York, NY, USA), microwave digestion system (Preeken EXCEl, Shanghai, China), multi-standard uranium and thorium solutions (Spex Certiprep Inc, Metuchen, NJ, USA) were all used in this work. The ultrapure water (18.2 MΩ/cm) was obtained from the Milli-Q System (Millipore, Billerica, MA, USA). Before measurement, ^209^Bi (Spex Certiprep Inc, Metuchen, NJ, USA) was added to all solutions as the internal standard. The reference materials of the National Institute of Standards and Technology (SRM 2783), International Atomic Energy Agency Standards (IAEA-312), and National Reference Materials of China (GBW-07103) were all applied to experimental assessment. All reagents containing hydrochloric acid (37% *w/w*, Beijing), nitric acid (69% *w/w*, Beijing), and hydrogen peroxide (30% *w/w*, Beijing) were of analytical grade except hydrofluoric acid (GR, Shanghai, China).

### 2.3. Sample Digestion

All samples were digested by the microwave system. The samples collected from the living area were digested by 1.5 mL of nitric acid, 1.0 mL of hydrogen peroxide, and 4.5 mL of hydrochloric acid. The digestion temperature was maintained at 150 °C for 5 min and 205 °C for 15 min at the maximum standard atmospheric pressure settings of 20 and 40 kPa, respectively. The samples collected from the occupational workplace were digested by 4.0 mL of nitric acid, 1.0 mL of hydrogen peroxide, 12.0 mL of hydrochloric acid, and 3.0 mL of hydrofluoric acid. The digestion temperatures were maintained at 120 °C for 20 min (20 kPa), 180 °C for 20 min (30 kPa), and 200 °C for 45 min (40 kPa). After digestion, the solution was heated to nearly dry. Then, the dryness solution was transferred into a capacity bottle, and the washing procedure was performed using ultrapure water. Finally, the volume of the solution was adjusted to 10 mL with ultrapure water. The digestion procedures of the blank and reference materials were prepared in the same manner.

### 2.4. Sample Analysis

All samples were determined using ICP-MS. The instrument parameters settings were taken from a published report [19]. The tuning fluid of the mass spectrometer was applied for quality correction and resolution calibration, and ^209^Bi was added to all solutions as the internal standard before measurement. Calibration curves for a series of uranium and thorium standard solutions (10, 100, and 500 ng/L and 1, 5, and 10 μg/L) were created for each analysis. The cleaning fluid during measurement was 2% (*v/v*) nitric acid. All of the testing samples were filtered with 0.22 μm of hydrophilic membrane before analysis by ICP-MS. The cellulose filter membrane reference (SRM 2783), the soil standard materials (IAEA-312), and the rock standards (GBW-07103) were all applied in the evaluation of the experimental process. The results obtained by using our procedure were all compared with the concentrations of known reference standard materials.

## 3. Results

### 3.1. Activity Median Aerodynamic Diameter Determination Experiment

**Figure 1:**
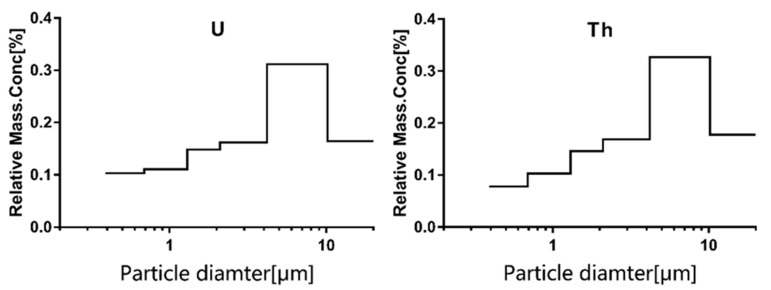
PM size distribution of uranium and thorium in atmosphere.

Here, 222 uranium and thorium contents, including six scales (0.39–0.69, 0.69–1.3, 1.3–2.1, 2.1–4.2, 4.2–10.2, and >10.2 µm), were obtained. The observed particle sizes of uranium and thorium and their corresponding relative mass concentration percentages are listed in Figure 1. The 50% cumulative frequency cutoff point was set in the range of 2.1–4.2 µm.

Researchers generally believe that the particle size distribution of radionuclide in the atmosphere has a log-normal distribution [20,21,22]. The particle size distributions of uranium and thorium are also assumed to have a log-normal distribution. Thirty-seven AMAD values of uranium and thorium were obtained on the basis of the log-normal distribution assumption in this study. The min, 25th, median, 75th, and max of the AMAD for the 37 samples are shown in Table 1. The average AMAD values of the uranium and thorium PM were 3.36 and 3.64 μm, respectively.

### 3.2. Estimation of Inhaled Effective Doses of Uranium and Thorium for Workers and the Surrounding Public

According to the natural abundance of uranium in the crust, the gram (g) unit of natural uranium was converted into Becquerel (Bq) by using the conversion factor of 1 g = 12.35 kBq of ^238^U, 0.57 kBq of ^235^U, and 12.38 kBq of ^234^U. The conversion factor of natural thorium was 1 g = 4.07 kBq of ^232^Th.

The protective mask used by the occupational population in the ore site is KN90, which means that 10.0% of the PM can penetrate the mask [23]. The occupational workers are assumed to have been involved heavy labor, and the inhalation volume during working time is 13.5 m^3^/day [24]. Also, all employees are assumed to have stayed in the surrounding living area except during working time, and the total inhalation volume is 13.3 m^3^/day during the non-working time of employees [24]. The activities of the residents of the surrounding living area are recognized as light labor, and the inhalation volume is assumed to be 22.9 m^3^/day [24]. According to the responses from the self-designed questionnaire, the median working time of the employees is 251 days per year and 8 h per day.

The dose coefficient is greatly affected by AMAD. However, only a few dose coefficients based on dispersed AMAD have been reported in the international commission on radiological protection (ICRP) reports [25], including 0.001, 0.003, 0.01, 0.03, 0.1, 0.3, 1.0, 3.0, 5.0, 10.0, and 20.0 μm. The AMAD in this study was assumed to be 5 μm for the occupational workplace [15] and 3 μm for the surrounding living area in accordance with Section 3.1. The dose coefficients were obtained from ICRP 137 [25]. Formula (1) [26,27] was applied to estimate the inhaled effective dose of uranium and thorium in the atmosphere. The calculated effective doses of uranium and thorium are listed in Table 2.

(1) $$E\left(\tau \right)=\left(1-\eta \right)\times C_{j}\times B\times e{\left(\tau \right)}_{j}$$

where

η—mask filtration efficiency, in which 90.0% is obtained for the workers in this study

$C_{j}$ —activity of radionuclide *j* in the air, Bq/m^3^

*B* —annual ventilation rate, m^3^/year

$e{\left(\tau \right)}_{j}$—dose coefficient for the inhalation of radionuclide *j*, S_V_/Bq

E(τ)—annual effective dose, Sv

## 4. Discussion

Natural uranium and thorium are the parent nuclei of most natural decay products and are ubiquitously in the crust where they are present at average concentrations of 2.8 and 7.4 μg/g [11]. In our study, hydrofluoric acid was necessary for digesting the PM for the workplace samples, but it was not obligatory for digesting PM in the living place specimens. The probable cause may be attributed to the different size distributions or chemical compositions of PM between the workplace and living area. The methods of digestion and measurement were assessed on the basis of their corresponding reference materials. Similar experimental procedures were previously reported by some studies [19,26,28,29,30,31].

AMAD is an important parameter when evaluating the hazardous effect of radionuclide in PMs on the human body [32]. PM is characterized as particles that dynamically impact each other and undergo sedimentation and diffusion [18]. Large PM is likely blocked by the nose, mouth, and lager respiratory tract and has low likelihood to reach the lung area. PM that enters the bronchioles of the lungs is the main hazard to the human body. The AMAD of natural radionuclides (^40^K, ^210^Pb, and ^7^Be) have been reported in the literature [15,20,21,22,33], but the AMAD of natural uranium and thorium is rarely investigated. The average AMAD values of natural uranium and thorium in the living area were estimated to be 3.36 and 3.64 μm, respectively. However, due to the certain limiting factors, such as the management policy and poor operability of occupational places, the determined AMAD of uranium and thorium needs to be further studied. According to the literature [18], when the AMAD of the inhaled nuclide is unknown, the AMAD for occupational workplaces is always assumed to be 5 μm. Given that the dose coefficient has been given on the basis of several various dispersed AMAD, the AMAD for the occupational population is 5 μm when the dose coefficient can be obtained. Meanwhile, the AMAD for the surrounding public was assumed to be 3 μm on basis of the fact that the AMAD of uranium and thorium was close to 3 μm in our study.

The measurement of inhalation can be performed using personal air samplers (PAS), which is generally known to be the most accurate method. However, this approach cannot be used routinely owing to several existing difficulties, such as the unwillingness of workers to wear PAS and the resulting low sampling volume. High-flow sampling equipment techniques can be used to complement these deficiencies.

The occupational place in our study only involved the areas where the physical processes of ore mining and beneficiation activities are being conducted. The uranium and thorium isotopes in our study area have natural abundance. The thorium in the natural environment exists almost entirely in the form of ^232^Th, while uranium exists in 99.275% of ^238^U, 0.720% of ^235^U, and 0.005% of ^234^U [34]. Therefore, when the inhaled effective doses of uranium and thorium were estimated, the natural abundance of the radionuclides of ^238^U, ^235^U, ^234^U, and ^232^Th were all considered.

In the experimental procedure, the possible components of the sample, including living area and occupational site, were analogically analyzed by using standard materials containing SRM 2783, IAEA-312, and GBW-07103. The measurement results were all consistent with the reference values, indicating that the measurement process could truly reflect the actual values. In the process of effective dose estimation, the following possible influencing factors were considered: AMAD, mask filtration efficiency, various residence times and dose coefficients of the workplace and the surrounding public, inhalation volume of different working environments, and so on. With this approach, the estimation of effective dose is expected to be close to the true value.

The median of the inhaled effective doses of uranium and thorium for the occupational workers ranged from 4.8 to 82.7 nSv/a and from 15.3 to 269.0 μSv/a, and the corresponding values for the surrounding public were 0.8 nSv/a and 2.3 μSv/a. The limited number of samples collected from the non-workplace in this study showed the consistent result with previous study [26]. The recorded data were all significantly less than the annual radiation dose induced by the natural source (2.4 mSv/a) [11] and lower than that in China (2.3 mSv/a) [35], but they are significantly higher than those for the normal living environment of China (1.206 μSv/a) [26].

## 5. Conclusions

The annual effective doses caused by inhaling uranium and thorium from typical radioactive RE ore were in the nSv and μSv magnitudes according to its concentrations in the atmosphere, respectively. However, the inhaled effective doses of uranium and thorium from the selected radioactive ore sites were lower than the doses induced by natural radiation sources. The obtained value was much higher than that of the normal living environment presented in other reports, especially for workers of ore sites. Significant attention should be given to the inhalation of uranium and thorium by workers and the surrounding public from associated radioactive ore sites in the future.

## Figures and Tables

**Table 1:** Descriptive parameters of AMAD for uranium and thorium PM.

AMAD	Min	*P*_25_	Median	*P*_75_	Max
Uranium	2.07	2.97	3.36	3.92	5.68
Thorium	2.37	3.27	3.64	4.01	6.84

*P*_25_ and *P*_75_ are the 25th and 75th percentiles, respectively.

**Table 2:** Effective dose of uranium and thorium inhaled by workers and the public.

Sample Spots	Uranium (nSv/a)			Thorium (μSv/a)		
	*P*_25_	Median	*P*_75_	*P*_25_	Median	*P*_75_
Surrounding public	0.8	0.8	1.4	2.3	2.3	3.9
Employees at spot 1	70.0	82.7	104.6	227.8	269.0	340.4
Employees at spot 2	8.1	17.0	41.8	26.0	55.1	135.7
Employees at spot 3	6.7	15.1	28.2	21.5	49.0	91.4
Employees at spot 4	2.9	4.8	13.2	9.2	15.3	42.5

*P*_25_ and *P*_75_ are the 25th and 75th percentiles, respectively.

## Data Availability

The data presented in this study are available on request from the corresponding author.
